# Sampling Scarab Beetles in Tropical Forests: The Effect of Light Source and Night Sampling Periods

**DOI:** 10.1673/031.011.9501

**Published:** 2011-07-26

**Authors:** Alejandra García-López, Estefanía Micó, Manuel A. Zumbado, Eduardo Galante

**Affiliations:** ^1^ Centro Iberoamericano de la Biodiversidad (CIBIO), Universidad de Alicante, San Vicente del Raspeig, 03080 Alicante, Spain; ^2^ Instituto Nacional de Biodiversidad (INBio), Santo Domingo de Heredia, Costa Rica

**Keywords:** cool white light trap, Costa Rica, Dynastinae, Melolonthinae, mercury-vapour light trap, Rutelinae, trap efficacy, ultraviolet light trap

## Abstract

Light traps have been used widely to sample insect abundance and diversity, but their performance for sampling scarab beetles in tropical forests based on light source type and sampling hours throughout the night has not been evaluated. The efficiency of mercury-vapour lamps, cool white light and ultraviolet light sources in attracting Dynastinae, Melolonthinae and Rutelinae scarab beetles, and the most adequate period of the night to carry out the sampling was tested in different forest areas of Costa Rica. Our results showed that light source wavelengths and hours of sampling influenced scarab beetle catches. No significant differences were observed in trap performance between the ultraviolet light and mercury-vapour traps, whereas these two methods caught significantly more species richness and abundance than cool white light traps. Species composition also varied between methods. Large differences appear between catches in the sampling period, with the first five hours of the night being more effective than the last five hours. Because of their high efficiency and logistic advantages, we recommend ultraviolet light traps deployed during the first hours of the night as the best sampling method for biodiversity studies of those scarab beetles in tropical forests.

## Resumen

Las trampas de luz han sido ampliamente utilizadas en el muestreo de la diversidad y abundancia de insectos, pero su eficacia en el muestreo de escarabeidos en bosques tropicales en función de la fuente de luz y del periodo de muestreo durante la noche no ha sido investigada. Se evaluó la eficacia de trampas con luz de vapor de mercurio, ultravioleta y blanca fría en la atracción de escarabeidos pertenecientes a las subfamilias Dynastinae, Melolonthinae y Rutelinae y el periodo de la noche más adecuado para llevar a cabo los muestreos en diferentes bosques de Costa Rica. Los resultados mostraron que las longitudes de onda de las luces utilizadas y el periodo de muestreo influyen en las capturas de estos escarabeidos. No se observaron diferencias significativas entre la eficacia de la luz ultravioleta y la de vapor de mercurio, mientras que ambos métodos capturaron significativamente más riqueza de especies y abundancia que la luz blanca fría. La composición de especies también varió entre métodos. Se encontraron diferencias entre los dos periodos de muestreo, siendo las cinco primeras horas de la noche más efectivas que las cinco últimas. Dada su elevada eficacia y ventajas logísticas, recomendamos el uso de trampas de luz ultravioleta durante las primeras horas de la noche como el mejor método de muestreo en estudios de biodiversidad de estos escarabeidos en bosques tropicales.

## Introduction

The current loss in biodiversity and degradation of natural habitats emphasize the need to take inventory of species richness and monitor changes in diversity. Sampling is the basis of studies documenting the spatial distribution of species or assessing changes in ecosystem structure, composition and function over time (Kremen et al. 1993; Heywood 1995; Humphries et al. 1995; Stork and Samways 1995; Yoccoz et al. 2001; Coscaron et al. 2009). For sampling the different taxa, it is essential to use the simplest and most effective method (Southwood and Henderson 2000) and since not all taxonomic groups are attracted in the same way to different capture methods, an adequate sampling method must be based on taxon-specific collecting procedures (Magurran 2004). Moreover, it has to be effective and easy to carry out to be able to be replicated in space and time.

Beetles are important components of the forest fauna due to their high abundance, diversity, and involvement in many ecological processes (Lawrence et al. 2000). For example, dung beetles (Coleoptera: Scarabaeidae: Scarabaeinae) are broadly recognized as a useful taxon for describing and monitoring patterns of biodiversity both in tropical and temperate areas (Allegro and Sciaky 2003; Nichols et al. 2007). Saproxylic and phytophagous beetles represent an important source of information in forest biodiversity studies. Species of Dynastinae, Melolonthinae and Rutelinae (Coleoptera: Scarabaeidae) are broadly represented in tropical forests and their tropic habits keep them closely linked to the ecosystem (Ritcher 1958). Adults of these subfamilies are mainly phytophagous, whereas the larval stages feed on roots or are saproxylic and play key roles in the ecosystem through wood decomposition and nutrient recycling (Ritcher 1958). For all these reasons the use of these groups of beetles is helpful as a tool for evaluating forestry biodiversity in tropical forests (Morón 2001, 2003).

Many different methods for collecting beetle species have been used for research purposes and inventories depending on their biology (Lobo et al. 1988; White et al. 1990; Cronin and Hayes 2000; Falach and Shani 2000; McIntosh et al. 2001; Missa et al. 2009). Light traps are assumed to be highly effective for trapping some groups of beetles with nocturnal activity, such as most Dynastinae, Melolonthinae and Rutelinae. Many studies focus sampling methods on these kinds of traps (Blomberg et al. 1976; Watson 1979; Hébert et al. 2000; Kato et al. 2000; Castro-Ramírez et al. 2003; Hirao et al. 2008; Wolda et al. 1998). Unfortunately, studies using light traps vary in light source, type of trap and period of sampling, which hampers comparison of results from different studies. Standardized sampling methods are essential for comparing species diversity and abundance patterns across different studies and sites. Commonly, studies on the efficacy of light traps focus their attention on the effect of the light trap wavelength (Carlson 1972; Walker and Galbreat 1979; Intachat and Woiwod 1999; Nabli et al. 1999), but the capture period during the night when the traps are operating is also an important parameter that influences insect catches (Mikkola 1972; Scalercio et al. 2009). In this way many groups of insects exhibit peak flight activity during twilight, particularly at dusk when temperatures tend to be higher than at dawn (Racey and Swift 1985). Restricting sampling to a period during only part of the night could improve the method by minimizing effort while maximizing obtained information (Scalercio et al. 2009).

This work analyzes the efficacy of three types of light trap equipped with three of the most commonly used wavelengths (see Carlson 1972; Intachat and Woiwod 1999; Kato et al. 2000; Nabli et al. 1999; Walker and Galbreat 1979) to sample three subfamilies of saproxylic and phytophagous Scarabaeidae (Dynastinae, Rutelinae and Melolonthinae) in tropical forests. Mercury-vapour lamp, cool white light and ultraviolet light trap catches from three forested ecosystems in Costa Rica were compared. The difference in the captured diversity between two different periods of the night, from 6 p.m. to 11 p.m. and from 12 p.m. to 5 a.m. was also investigated. The main questions were (1) What is the most efficient light source in terms of abundance and species richness? (2) What are the effects of sampling methods on the species composition of trap catches? (3) What is the most efficient period of the night that allows us to reduce the sampling effort?

## Materials and Methods

### Studied Group

We selected the subfamilies Rutelinae, Melolonthinae and Dynastinae. The subfamily Melolonthinae is world-wide in distribution; adults of most genera feed extensively on the foliage of trees and shrubs, but some adults attack flowers or fruits. Larvae are subterranean feeders on roots and many of the most injurious species of the family Sacarabaeidae belong to this group. The subfamily Rutelinae reaches its greatest species richness in the neotropical region. Adults of this subfamily are mainly leaf-feeding beetles while larvae could be root-feeders (such as in the Anomalini tribe) or feed on decaying wood (such as in Rutelini). The subfamily Dynastinae is mainly saproxylic. However, some of them are pests of crops due of the phytophagous habit of the larvae (Ritcher 1966). The adults of most of the species of the three subfamilies lie hidden during the day carrying out their alimentation, reproduction and dispersion activities during the night (Morón 2004). This fact optimizes the capture through the light traps like those used in this work (see Sampling Methods).

### Sampling Methods

Treatments consisted of three different light sources: mercury-vapour light (MVL), ultraviolet light (UVL) and cool white light (CWL). MVL (CEW, W39KB-175) consisted in a 175 W lamp with a broad spectrum with major peaks at 253.7 nm, 365.4 nm (1-line), 404.7 nm (H-line), 435.8 nm (G-line), 546.1 nm, and 578.2 nm. UVL (Philips, TL-D 18W/108 Black light blue) and CWL (Osram, L 18W/765 Cool Daylight) were 18 W fluorescent tubes of length 60 cm. UVL has major peaks in the ultraviolet region at around 365 nm. The CWL has major peaks around 440 and 580 nm, with some ultraviolet light.

**Figure 1.**
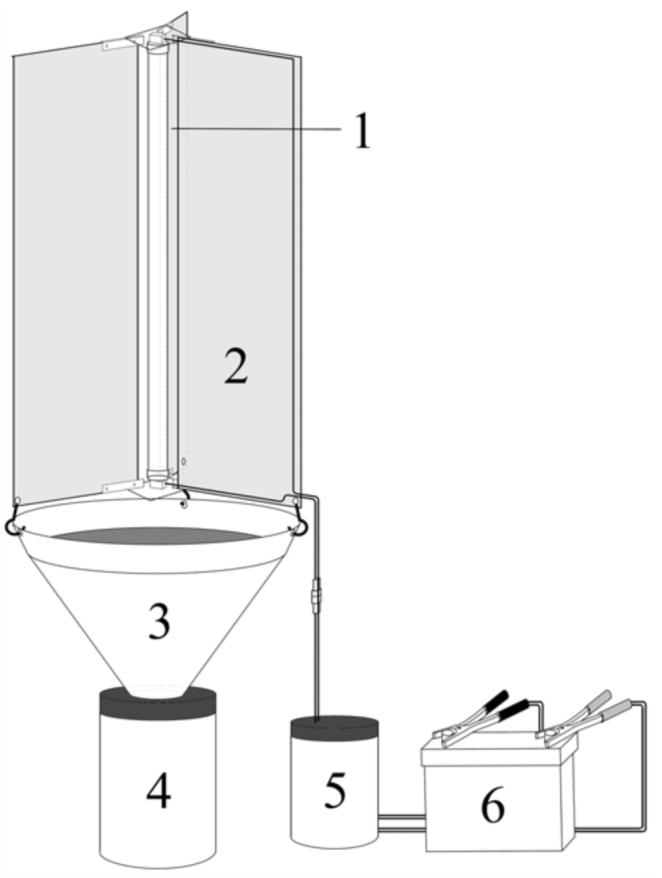
UVL and CWL traps. (1) Light source, (2) transparent plastic sheets around the light source, (3) funnel, (4) collector bottle (5) bottle protecting the electrical components (ballast and timer) and (6) battery. High quality figures are available online.

In the MVL trap (BioQuip, 2818) the light source reflects onto a white vertical screen. The light is powered by a generator or directly connected to the electrical grid. The light is switched on or switched off manually and the presence of investigators is also necessary for the sampling of specimens. On the contrary, the UVL and CWL traps are fed by a lightweight battery (35 Amp, 12 V) and work completely alone. They are switched on or switched off automatically and the specimens are captured without the presence of investigators (Figure 1).

UVL and CWL traps consisted of the light source, three transparent plastic sheets around the light source against which the insects crash when they are attracted by the light, a funnel in the base of the sheets that directs the specimens to the collector bottle, a bottle protecting the electrical components (ballast and a timer that switches on and switches off the light at the chosen hour) and the battery that feeds the light and the timer (Figure 1). Traps were hung on a tree branch at approximately 1.5 m above the floor and were protected from the rain by a transparent plastic roof of around 1.5 m^2^. This model of trap can be adapted to ecosystems without the presence of trees with the use of a tripod to support the structure of the trap.

### Study Areas and Scarab Beetle Collection

Specimens were collected from five sampling sites in different forest areas of Costa Rica. These forests were situated at different altitudes and had different ecological characteristics that allowed for testing the performance of the traps under different conditions. Data for analysis of efficiency of the different light traps correspond to sampling with MVL, CWL and UVL traps at the sites El Copal, Heliconias and El Pilón (Table 1). Data for the analysis of catches in different periods of the night correspond to sampling with UVL traps at the sites La Esperanza, Tapantí and El Pilón (Table 2).

In the analysis of the efficiency of the three light trap methods, each site was equipped with an identical set of traps. At each site, two MVL traps, two CWL traps and two UVL traps were used. Traps worked simultaneously during five hours from 6 p.m. to 11 p.m. on consecutive nights. In the analysis of the different sampling period throughout the night two UVL traps were used at each site. These traps operated simultaneously for two periods of five hours from 6 p.m. to 11 p.m. and from 12 p.m. to 5 a.m. All the samplings were carried out during the days around the dark moon to avoid differences in the effect of the lunar cycle which can affect nocturnal insect activity and catch ability (Bowden 1973, Brown and Taylor 1971, Nowinszky et al. 1979). After each period of sampling, the traps' contents were removed and stored for later identification. Specimens were deposited in public collections of the Instituto Nacional de la Biodiversidad, Costa Rica (INBio) and Colección Entomológica de la Universidad de Alicante, Spain (CEUA).

### Data Analysis

Variation in species richness and abundance per sampling day among the three sampling methods was evaluated using a Kruskal-Wallis test and Bonferroni post hoc test. Comparisons between light traps were performed for the overall data and separately for each sampling site to test if the pattern found was shared. The null hypothesis tested was that all light sources were equally attractive to the studied beetles. The same comparison between the three traps was performed for the total species richness and abundance sampled. Kruskal-Wallis tests were done with STATISTICA (StatSoft 2007). The light traps were also compared by calculating the percentages made up by each subfamily of beetles in the total catch for each light trap, totalled over all sampled nights.

Complementarity between methods was investigated by calculating the variation in species composition between the three light traps using the Bray-Curtis similarity measure of presence and absence matrices (Bray and Curtis 1957). This analysis calculates the proportion of all species collected by two methods that were captured by only one method. This value varies from 100 (both methods share all species) to 0 (methods have no species in common). Analysis of similarity was done with PRIMER (Clarke and Gorley 2006). The light traps were also compared by analyzing the distribution of unique and shared species for and between light traps.

**Figure 2.**
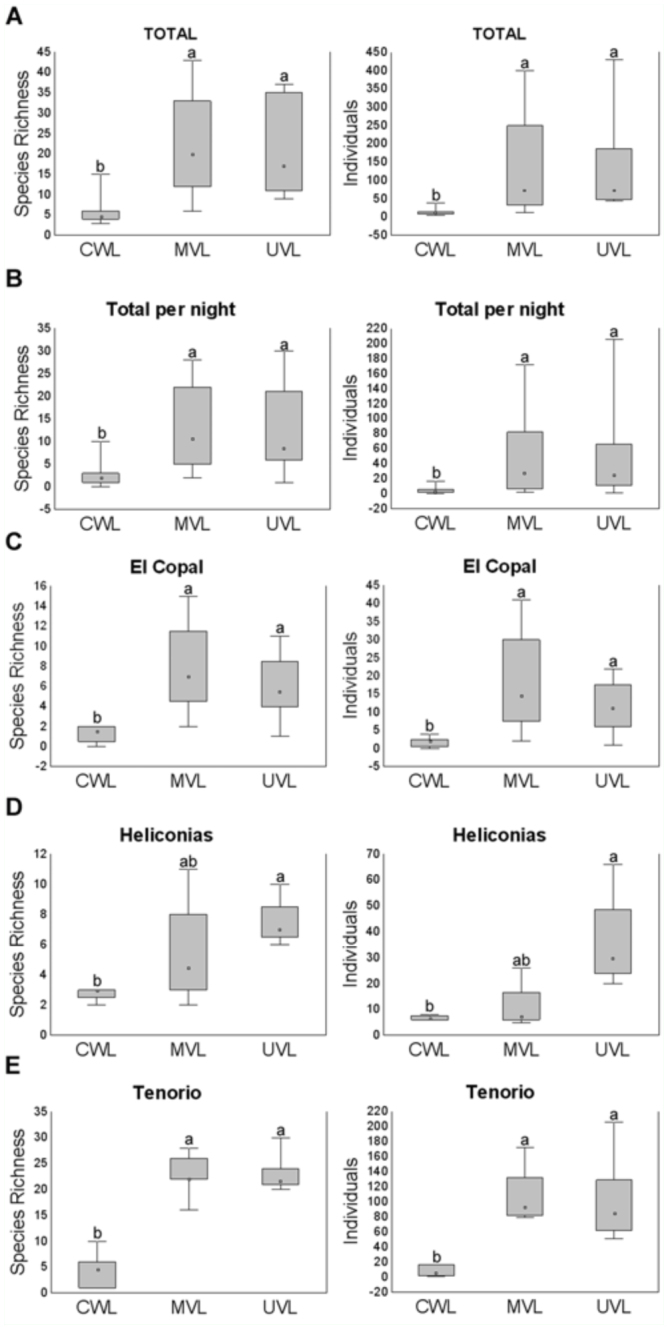
Attraction patterns towards light traps based on species richness and abundance for the total of samples (a), expressed per trap-day for each sampling site (c, d and e) and for the overall data (b). A median (bar), quartiles (box), a minimum and a maximum (whisker) and outliers of species richness or abundance for each sample are represented as box-whisker plots. Comparisons are based on Kruskal—Wallis and subsequent post-hoc test; traps with the same letter indicate no difference in the value of species richness or abundance. High quality figures are available online.

Similar analyses using the Kruskal-Wallis test and the Bonferroni post hoc test were conducted to compare the number of species and individuals captured during the two periods of sampling, both for the total species richness and abundance sampled and for each subfamily separately. The null hypothesis tested was that the two sampling periods did not impact the catch. Also, the variation of the unique and shared species distribution between the two sampling periods was observed.

## Results

A total of 1783 individuals belonging to 97 species and 25 genera were collected during the sampling to test the efficiency of the three different light traps (Appendix 1). During the different sampling period test, 922 specimens of 76 species and 23 genera were collected (Appendix 2).

Catch rates expressed per trap-day were significantly different among methods, both for each sampling site and for the overall data. Total beetle species richness and abundance also varied significantly between traps (Tables 3 and 4). Post hoc tests showed that in all the cases, with the exception of the site at Heliconias, there were no significant differences between the effectiveness of the MVL trap and the UVL trap, whereas the catches from the CWL trap were significantly lower (p < 0.01) (Figures 2A, 2B, 2C and 2E). In the case of Heliconias field site, there are no significant differences between the MVL trap and the CWL trap (Figure 2D), probably due to a lower N sampled (Table 3).

The percentages of richness and abundance for each subfamily captured by each method were similar to those found when we analyzed the three subfamilies together. In all the cases, the CWL trap was the method with the lowest effectiveness in the capture of the three subfamilies (Table 5).

The assemblage of beetle similarity also varied between methods. Species composition among the catches of the MVL trap and the UVL trap was highly similar (Bray-Curtis Index = 76.2%), while the similarity between the catches of the MVL trap and the CWL trap (Bray-Curtis Index = 51.5%) and between the catches of the UVL trap and the CWL trap (Bray-Curtis Index = 52%) was lower.

**Figure 3.**
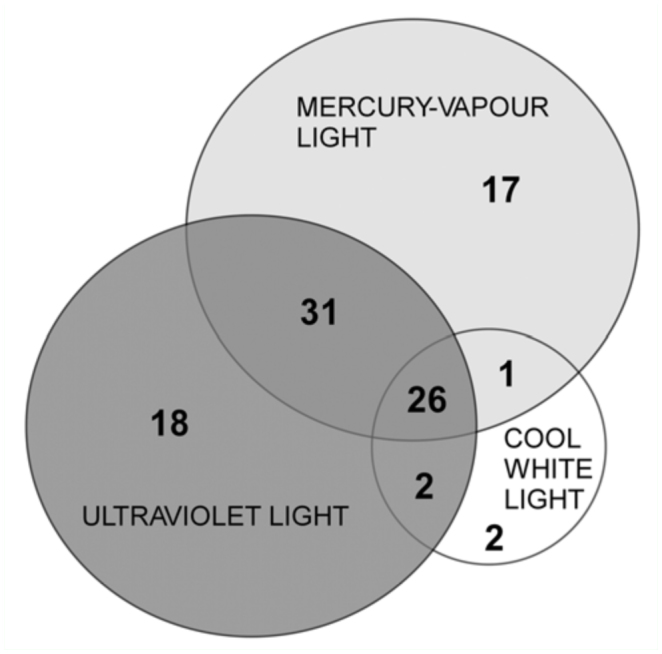
Venn diagram showing species caught in the three trap types. High quality figures are available online.

The distribution of unique and shared species was broadly variable depending on the capture method. Only 26 species (26.8% of total sorted) were collected by all three sampling methods. Both the MVL and the UVL trap produced a high proportion of unique species and together they contain 97.9% of the total species collected. However, two species were collected solely by the CWL trap (Figure 3).

**Figure 4.**
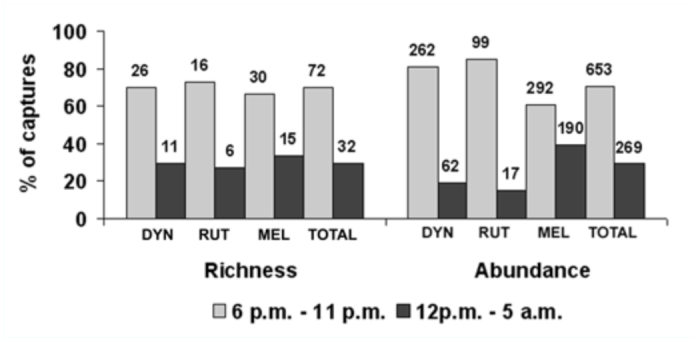
Total abundance and species richness in both studied periods for the overall data and for each subfamily separately. High quality figures are available online.

Species richness and abundance also varied significantly between sampling periods, both for the overall data and for each subfamily separately, with the exception of Rutelinae where no significant differences were found (Table 7). This absence of significant difference in the Kruskal-Wallis analysis for Rutelinae is probably due to the high variation in species richness and abundance found among the sampled sites and not to a real similarity between the diversity captured during both periods (Figure 4). When significant differences happened, post hoc tests showed that, in both species richness and abundance, the first period of the night is significantly more effective in the capture of the studied group (p < 0.05), representing high percentages of capture for the total catches and for each subfamily (Figure 4). Regarding composition, the first sampling period (6 p.m. – 11 p.m.) produced 57.9% of unique species (N = 44) whereas only 5.2% (N = 4) appeared during the second sampling period (12 p.m. – 5 a.m.) and were represented by only one specimen. The percentage of species collected during both of the sampling periods was 36.8% (N = 28).

## Discussion

Our results showed that the effectiveness of the MVL and the UVL traps in terms of sampled species richness and abundance were similar and significantly higher to that of the CWL trap (Figure 2). This study confirms the existence of different preferences among insects for specific light sources (Blomberg et al. 1976, Walker and Galbreat 1979, Nabli et al. 1999, Fayle 2007) emphasizing the importance of taxon-specific studies to define effective and easy to standardize sampling methods.

For scarab beetles studied, MVL and the UVL traps appeared equally useful for biological monitoring of the group, whereas the high values of complementarity between them (Figure 3) indicates that for full species inventories, a combination of both approaches is recommended.

The best period of the night to carry out the sampling also depends on the taxonomic group because it is influenced by the flying behaviour of the species and must be determined in each case. For scarab beetles, our results showed that sampling between 6 p.m. and 11 p.m. was most effective (Figure 4, Table 6). This coincides with other studies where authors found a decrease in diversity throughout the night (Mikkola 1972; Scalercio et al. 2009). Nevertheless, as there is no information about the activity of these scarabs during the night and the way they are attracted to light, it could be that the second period catches were affected by those of the first one. Specific studies are needed to answer these questions. However, when it comes to minimizing effort and maximising information, our results indicated that a sampling during the first five hours of the night produces a high percentage of the total diversity found over a complete night (Figure 4).

In spite of the similar efficacy of the UVL trap and the MVL trap, the UVL trap has important advantages. Because it is an automatic trap, it does not require the presence of the investigator, nor is its efficacy affected by the number of people at the trap and their experience. Thus, the UVL trap allows better standardization of protocols. It is fed by a small battery, whereas the MVL trap needs either a back-up generator or a connection to an electrical grid. Hence, when working away from main power, the sampling is more difficult because it is necessary to carry a heavy generator. Moreover, the UVL trap can be quickly and easily set out in the field, allowing high spatial replication for habitat comparisons and permitting rigorous statistical analysis.

Because of their high efficiency, possibilities for standardized sampling, easy transport and capacity to work without the presence of the investigator, we conclude that the use of UVL traps during the first five hours of the night is the most practical sampling method for studies of saproxylic and phytophagous scarab beetles in tropical forests.

**Table 1.**
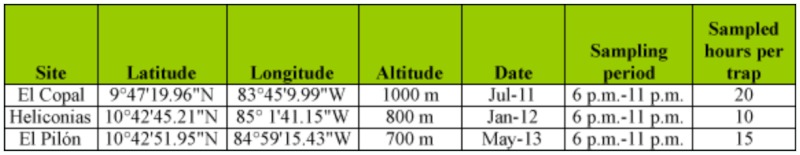
Sampling sites for the analysis of the efficiency of the different light traps.

**Table 2.**
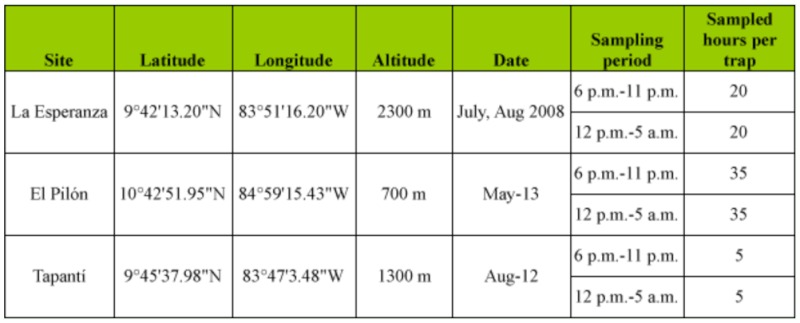
Sampling sites for the analysis of catches in different periods of hours.

**Table 3.**
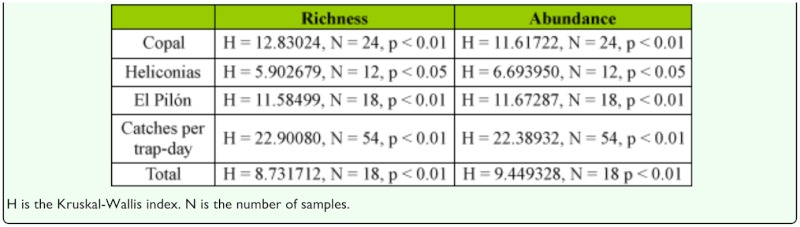
Variation in species richness and abundance results among the three sampling methods using a Kruskal-Wallis test.

**Table 4.**
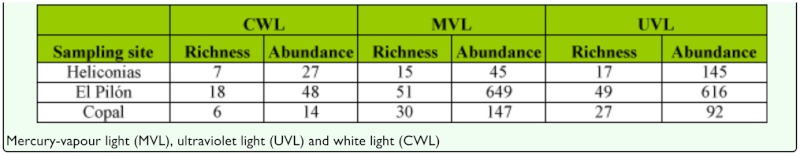
Species richness and abundance trapped by the three light traps in each sampling site.

**Table 5.**
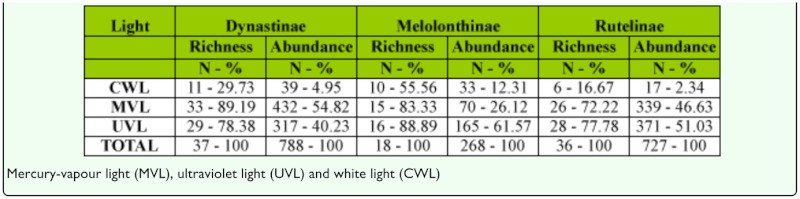
Species richness, abundance and percentages of capture of each subfamily trapped by the three light traps.

**Table 6.**
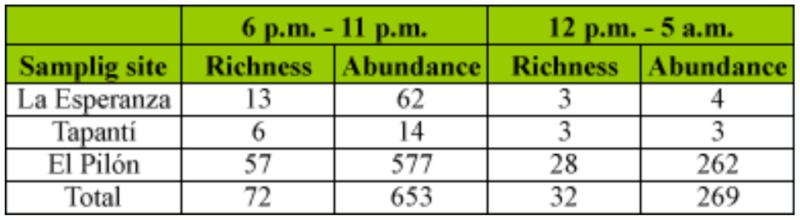
Species richness and abundance per sampling site for each sampling period studied.

**Table 7.**
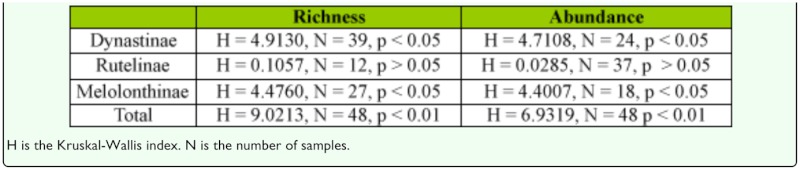
Variation in species richness and abundance results among the two sampling periods, for the overall data and for each subfamily separately, using a Kruskal-Wallis test.

## Figures and Tables

**Table 1.**
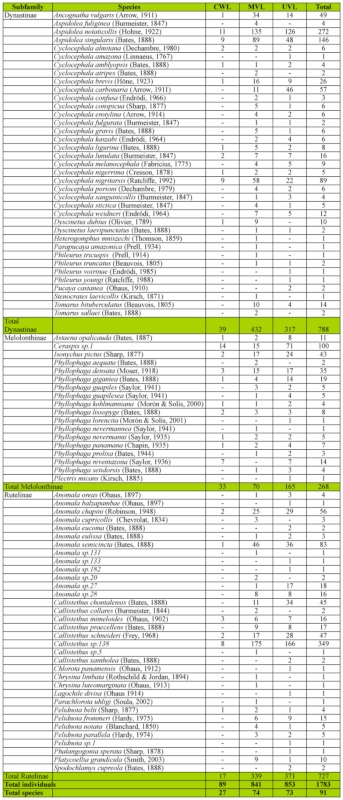
Individuals of Scarab beetle species collected during the samplings for the analysis of the efficiency of the three light traps (MVL: mercury-vapour light; UVL: ultraviolet light; CWL: white light).

**Table 2.**
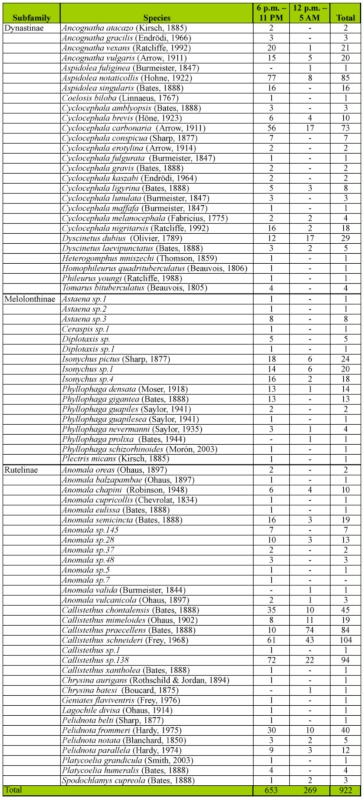
Individuals of Scarab beetle species collected during the samplings for the analysis of the different trap operating period throughout the night.

## Abbreviations

CWL,: cool white light;
MVL,: mercury-vapour light;
UVL,: ultraviolet light
